# Investigating association between second trimester maternal serum biomarkers and pre-term delivery

**Published:** 2013-02

**Authors:** Zahra Sehat, Azita Goshetasbi, Mehdi Taheri Amin

**Affiliations:** 1 *Avicenna Research Institute, Reproductive Biotechnology Research Center, Tehran, Iran.*; 2 *Department of Family Health Iranian In States for Health Sciences Research, Tehran, Iran.*; 3 *Department of Laboratory Sciences, Nilou Medical Laboratory, Tehran, Iran.*

**Keywords:** *Preterm Birth*, *Quadruple*, *Alpha-fetoprotein*, *Human Chorionic Gonadotropin*, *InhibinA*, *Non-conjugated estrogen*

## Abstract

**Background: **Considering the effect of preterm delivery in morbidity and mortality of newborns, its precaution and prevention is so important.

**Objective:** To investigate the association between second trimester maternal serum biomarkers (Human Chorionic Gonadotropin, Alpha-fetoprotein, Non-conjugated estrogen, Inhibin A) and pre-term delivery.

**Materials and Methods:** This is a historical cohort study that has been performed for 700 pregnant women, clients of Nilou Lab in the second trimester of pregnancy to take the Quad Marker test between March to September 2008. The information of mothers having required conditions to enter to study has been registered and after delivery, they called again to be interviewed. These data sets using statistical tests: chi-square test and Roc Curve was analysis.

**Results:** There is a direct relationship between preterm delivery and increase of Alpha-fetoprotein (p=0.011) and inhibin A (p=0.03) serum level and. Also, there is an inverse relationship between the non-conjugated estrogen (p=0.002) serum level and preterm delivery. Moreover, there is not any relationship between the increase human chorionic gonadotropin (p=0.68) serum level and preterm delivery.

**Conclusion:** The increase in the Alpha-fetoprotein and Inhibin A and decrease in Non-conjugated estrogen serum levels in the second trimester of pregnancy lead to enhance the probability of preterm delivery. Moreover, if the current study is done with higher samples and different sampling environment, it may have different results.

## Introduction

Preterm labor is one of the major health problems which is occurred and after major congenital malformations and mortality, neonatal morbidity is operating. Due to the economic costs of mental death at community level to be reduced, it is important to predict and prevent premature birth and infant mortality rates (1-3). 

Each year millions of children worldwide are born premature. Almost 75 percent of the prenatal morbidity and mortality is preterm delivery (4-6). Prevalence of preterm delivery in 2004 was about 12.5% in the United States (7). Since stopping the process of starting labor with preterm delivery has been associated with less success, today, researchers predict the possibility of preventing preterm labor .The first step to prevent preterm delivery and predict it in this early detection and treatment of women at risk during their prenatal care is part of the main objectives (8-11). 

Increased incidence of preterm labor is a significant thread in the whole world. In Western countries 6-10% of all births in 2007 were preterm, and more than two-thirds of all deaths during prenatal period and 75-80% of all deaths in babies was due to premature delivery (11-13). The incidence of preterm delivery before 37 weeks is approximately 7-11% and before 34 weeks it is 3-7% of all pregnancies (14). 

Despite much research and many advances in prenatal care, preterm birth rate has increased slightly in recent years. This increase could be due to higher prevalence of multiple deliveries following the use of ovulation drugs, increased obstetric intervention, and increased stress, progress in the discovery of preterm deliveries, the use of artificial reproductive techniques, and increased use of ultrasound to estimate gestational age.

Different methods such as biological and demographic factors, serum markers and cervical changes have been studied for screening to predict preterm delivery; also, during a study level of salivary estradiol and its association with preterm delivery has been investigated (14-16). Each of them has disadvantages, advantages, features, and its certain time to do. But, none of them has required sensitivity, specificity and positive predictive value.

According to recent studies on maternal serum markers in certain conditions, such as premature delivery and preeclampsia during pregnancy, we decided to evaluate the possible role of biochemical markers in the second trimester of pregnancy as a reliable test to predict preterm delivery (17, 18). In the screening test of Quadruple marker four biochemical markers (Human Chorionic Gonadotropin (HCG), Alpha-fetoprotein (AFP), Non-conjugated estrogen (UE3), Inhibin A) are measured in maternal blood in the second trimester. This study, review the value of maternal serum biochemical markers (HCG, AFP, UE3, Inhibin A) to predicts the incidence of preterm labor.

## Materials and methods

In a historical Cohort Study, pregnant mothers i.e. clients of Nilou Lab screened in the second trimester of pregnancy between March 2008 to September 2008, with Quad Marker were investigated for the relationship between serum levels of biochemical markers and the incidence of preterm delivery.

These individuals were selected with consideration of the following conditions:

-Singleton pregnancy

-No maternal infection or chronic systemic diseases including liver, heart or kidney disease, diabetes, lupus and etc. 

-BMI <30

- detectable fetal abnormalities

- detectable intrauterine death

-No maternal addiction to cigarettes, alcohol and drugs

-No diagnose of placental abruption premature

-No diagnosis of premature rupture of membranes

The mothers with higher risk of preterm delivery i.e. mothers having one of specifications like fetus with down syndrome, twin pregnancy, genetic or chromosomal disorder, history of preterm delivery, history of abortion and maternal diseases such as diabetes mellitus and chronic hypertension, addiction to cigarette, alcohol or opiate were excluded. Those who have entered into the study were selected and primary data were recorded and in the second trimester of pregnancy they were tested. Then after the birth of study population, all other information were taken by telephone, after obtaining verbal consent from them. Then with the help of appropriate statistical tests the relationship between the biomarker and the outcome of pregnancy were examined.

Between weeks 14-20 for Quad Marker, testing of 3^CC^ maternal blood samples was done. The blood samples were kept in the refrigerator for 24 hours at 2-8 degrees Celsius in order to separate plasma from serum. Then four biomarkers (HCG, AFP, UE3, Inhibin A) were measured. AFP and HCG were tested by Elecsys system using Electrochemilominescense Cobase cholera kits separately, UE3 and Inhibin A were measured by ELISA. Using UE3 DEMEDITEC Free Esteriol ELISA kit and DSC-10-28100 Active Inhibin A ELISA was evaluated.

To determine the cut-off point for the different biomarkers, the first false positive detection rate for the five-points 0.5, 0.75,1, 1.25 and 1.5 and the percent detection rate and also false positive percentage in the population studied has been determined. Two graphs, one depicting Detection rate versus cut off and other representing percentage of detection rate versus percentage of false positive. In the second graph, the point who has the minimum slope has been determined and the suitable cut off value is obtained from the first graph. This procedure has been repeated for all four markers and cut-off points 

The important note to choose a cut off is that MOM of choice with minimal false positive rate has the maximum sensitivity in this study; maximum 30% false positive has been placed at the base.

**Statistical analysis**

Data have been entered to SPSS 16.0 software and analyzed using the statistical and analytical tests like Roc Curve to obtain the cut-off point. Chi Square test was used to examine the relationship between preterm labor and biomarkers.

## Results

This study was conducted on 1,000 cases. In total 300 of them were excluded for reasons such as twin pregnancy, preeclampsia, placental abruption, PROM, detectable abnormalities in the fetus, risk of gestational diabetes or not willing to cooperate. At the end of the study population were 700 cases. In this study average age of mothers was 26.17±4.72 years and mean duration of pregnancy were 38.4±1.27 weeks. 7.3% of mothers have preterm labor and 93.7% have full term delivery. 

Between serum levels of AFP (p=0.011), Inhibin A (p=0.030) and preterm delivery direct relationship was seen. Relationship between redacted. Serum levels of UE3 (p=0.002) and preterm labor was positive. Relationship between preterm delivery and HCG (p=0.683) wasn't observed.

**Table I:** Demographic characteristics of the case studied

		**Preterm delivery ** **N (%)**	**Term delivery ** **N (%)**	**p-value**
Maternal age (year)				
	15-25	9 (16.98)	104 (16.07)	0.15
	26-30	22 (41.50)	255 (39.41)	
	31-35	22 (41.50)	281 (43.43)	
	>35	0 (0)	7 (1.08)	
First trimester maternal BMI (Kg/m^2^)				
	<25	15 (47.16)	340 (52.55)	0.38
	25-30	19 (35.84)	237 (36.63)	
	>30	9 (16.96)	70 (10.81)	
Job				
	Housewife	37 (69.81)	456 (70.47)	0.91
	Employed	16 (20.18)	191 (29.52)	
Number of previous pregnancies				
	>2	49 (92.46)	600 (92.73)	0.96
	<2	4 (7.54)	47 (7.26)	
History of abortion^*^		11 (20.76)	126 (19.7)	0.80
History of chronic illness^**^		7 (13.20)	60 (9.27)	0.73
History of preterm delivery^***^		13 (24.52)	39 (6.027)	0.90

BMI: Body Max Index.

*Termination of pregnancy by the removal or expulsion from the uterus of a fetus or embryo prior to viability.

**Maternal diseases such as diabetes mellitus and chronic hypertension.

***Delivery before 37 week of pregnancy.

**Table II:** Relationship between the occurrence of preterm delivery and levels of mother's serum biomarkers in second trimester of pregnancy

	**Standard error**	**p-value**	**Confidence interval**	**Curve**
HCG	0.41	0.74	0.567-0.47	0.4870
AFP	0.43	0.11	0.652-0.481	0.567
Inhibin A	0.43	0.22	0.46-0.63	0.55
UE3	0.37	0.00	0.465-0.319	0.392

HCG: Human Chorionic Gonadotropin.

AFP: Alpha-fetoprotein.

UE3: Non-conjugated estrogen*.*

**Table III:** Frequency of mothers serum levels of Quad marker second trimester of pregnancy

**Cut off (MOM)**	**Sensitivity**	**Specificity**	**PPV**	**NPV**
UE3<0.8	20.8%	57.79%	40.7%	59.3%
Inhibin A ≥1.5	20.8	89.2	11.6	88.4
HCG> 1.25	26.4	70.9	28.9	71.1
AFP>1.4	28.3	85	16	84

MOM: Multiple of the median.

PPV: Positive predictive value.

NPV: Negative predictive value.

**Table IV:** Frequency of Positive and Negative Quad marker test in the second trimester of pregnancy

**Quad marker**	**Preterm delivery**		**Delivery on time**		**Total**	
	** No.**	**%**	**No.**	**%**	**No.**	**%**
Positive^*^	35	66.03	435	67.23	470	67.1
Negative^**^	18	33.96	212	32.76	230	32.9

P-value=0.52

* Positive Quad marker test = (Cut off (MOM)) UE3<0.8, Inhibin A ≥1.5, HCG> 1.25, AFP>1.4

** Negative Quad marker test = (Cut off (MOM)) UE3>0.8, Inhibin A <1.5, HCG< 1.25, AFP<1.4

**Figure 1:**
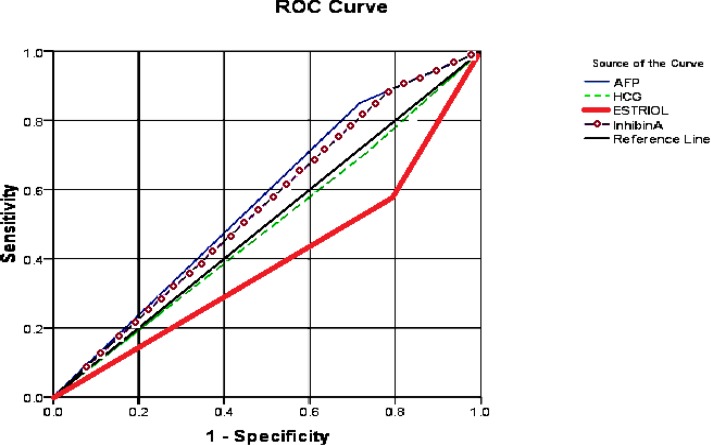
Relationship between the occurrence of preterm delivery and levels of mother's serum biomarkers in second trimester of pregnancy.

## Discussion

This study determined the level of maternal serum biochemical markers (AFP, HCG, Inhibin A, UE3) in the second trimester of pregnancy and its association with preterm labor. 700 pregnant women according to inclusion criteria were investigated. In this study the normal level of AFP maternal serum with MOM^1^≤1.4 and normal level InhibinA with MOM≤1.5, HCG with MOM≤1.25, and UE3 with MOM≥0.8 have been considered. 7.3% of mothers in this study had preterm labor. 

In other studies similar prevalence has been reported (12-17). In a study, that was conducted by Kim and colleagues, unlike the results obtained in this study. There was relationship between the incidence of preterm delivery and lower maternal serum levels of UE3 in the second trimester of pregnancy (8). In the Kim’s study which was conducted in 2000, 1096 mothers in their second pregnancy trimester have been screened regarding AFP, HCG and UE3 level. Individuals with levels of AFP and HCG having MOM> 2 have been excluded from the study to investigate more accurately impact of reduced UE3 level in the consequences of pregnancy. Two groups of people with MOM ≤0.75 levels and the level of UE3 MOM >0.75 have been divided and adverse consequences of pregnancy have been studied. But, in terms of incidence of preterm delivery, there was no difference between two groups.

Levels of UE3 in fetal and maternal serum depend on cooperation of placental steroids and availability of DHEA by the fetal adrenal. With the reduction in uterine blood flow, the pair is associated with reduced levels of UE3.

Thus, decreased levels of UE3 in the second trimester of the embryos or placental dysfunction can be seen, and in these cases fortunate pregnancy is not expected. In this study, cases with abnormal levels of AFP and HCG have been eliminated. While studies have shown that reduced levels of HCG and UE3 lead to increased levels of .But, in this study, UE3 levels have been measured without excluding individuals with abnormal levels of AFP and HCG. 

According to Dugoff and colleagues in 2005 a direct relationship has been seen between increasing maternal serum of AFP in the second trimester of pregnancy and the incidence of preterm delivery (p=0.047). Similarly, a direct relationship between increase in maternal serum of Inhibin A in second trimester of pregnancy and the incidence of preterm delivery has been seen (p=0.04) (17). The same results for AFP and Inhibin A have been optioned in the present research. 

In the Dugoff ‘s cohort study that 33,145 pregnant women take the Quad Marker test in the second trimester of pregnancy, relationship between the four components of Quad Marker with adverse pregnancy outcome has been evaluated. Such a result indicates that people with abnormal levels of biomarker are in a modest but significant risk of undesirable consequences of pregnancy; But, if the risk of having abnormal levels of two or more biomarker increase. For example, risk of preterm delivery in the case that only AFP level has been measured with MOM ≥2, is Odds Ratio: 1.76; but in case that AFP and Inhibin A levels has been measured with MOM ≥2, the risk is OR: 4.19. In case of AFP and Inhibin A, have been measured with MOM ≥2 and HCG and UE3 with MOM <0.5, risk is OR^1^: 4.05. 

In this study, sensitivity and positive predictive value test will be decrease, in simultaneous use of multiple biomarkers. On account of the fact that screening tests along with the sensitivity and positive predictive value, negative predictive value rates and Specificity can also be important to have an acceptable level of specificity for high levels of sensitivity. 

Based on the study performed in 2003, Duric *et al* assess the relationship between serum levels of three markers AFP, HCG and UE3 in 15-22 weeks of pregnancy with the consequences of pregnancy by a historical cohort method on 2384 pregnant women in Croatia. Their result was contrary to recent research results. They reported that increasing maternal serum HCG MOM ≥2.2 in the second trimester of pregnancy is associated with the incidence of preterm delivery (OR=2.5, p<0.05). This study compared with the presented study, has higher level of HCG Cut off that more at achievable in a different environment and more likely with higher sample numbers. Also, like this study, there is relation between reduced levels of UE3 in maternal serum in the second trimester of pregnancy and preterm labor relations (OR: 2.2, p<0.05) (18). 

In a study of Lalooha and colleagues in Iran 2005 performed, like the presented study the maternal serum AFP level increased in mothers with preterm labor has been observed more (p<0.05) (19). Therefore, it can be concluded that the AFP is a valuable biochemical marker to predict preterm labor.

Although AFP, Inhibin A and UE3 can predict the incidence of preterm labor, but due to the high cost of this screening test, usually not all of mothers are willing to do it. However, this point should be mentioned that these costs can spend a lot of psychological consequences and financial costs of preterm birth, fetal abnormalities and disease for individuals, families and communities to prevent. For this purpose, parental education about the benefits of screening tests must be performed during pregnancy is important. 
